# Analysis of central corneal thickness in systemic lupus erythematosus

**DOI:** 10.3389/fmed.2025.1545415

**Published:** 2025-02-28

**Authors:** Juan David Saldaña-Garrido, Mario Cantó-Cerdán, Vicente Francisco Gil-Guillén, María Luisa Alfaro-Beltrá, Francisca Sivera

**Affiliations:** ^1^Department of Ophthalmology, General University Hospital of Elda, Alicante, Spain; ^2^Department of Clinical Medicine, School of Medicine, Miguel Hernández de Elche University, San Juan de Alicante, Spain; ^3^Vissum Miranza, Alicante, Spain; ^4^Department of Investigations, General University Hospital of Elda, Alicante, Spain; ^5^Department of Rheumatology, General University Hospital of Elda, Alicante, Spain

**Keywords:** systemic lupus erythematosus, central corneal thickness, pachymetry, optical coherence tomography, glaucoma, hydroxychloroquine

## Abstract

**Introduction:**

Systemic lupus erythematosus (SLE) is a chronic autoimmune disease with ocular involvement in up to 30% of cases. Due to its type I collagen composition, the cornea is particularly susceptible to thinning due to immune-complex deposition. A reduced central corneal thickness (CCT) is clinically relevant in glaucoma, where a thinner CCT increases glaucoma risk and in refractive surgery planning. Previous studies on CCT in SLE are limited due to methodological heterogeneity, technology use, inclusion criteria, and sample size, resulting in conflicting findings. This study aims to evaluate and compare the mean CCT values between patients with SLE and healthy controls.

**Materials and methods:**

This cross-sectional study assessed mean CCT in 71 participants, 36 patients with SLE and 35 age- and sex-matched healthy controls, recruited from ophthalmology consultations. Participants with other risk factors for corneal thinning were excluded. A pilot study estimated a sample size of 34 participants per group. After confirming concordance using the Kappa index, one randomly selected eye per participant was included. CCT was measured using Zeiss HD Cirrus 5,000 optical coherence tomography. Correlation analysis was conducted using Spearman’s Rho coefficient, while a Loess regression was performed to visualize both linear and non-linear trends. Multivariate linear regression assessed the relationship between CCT, SLE, and other variables.

**Results:**

Patients in the SLE group exhibited significantly thicker CCT than controls (536.44 ± 39.91 μm vs. 517.57 ± 29.62 μm, *p* = 0.014). Intraocular pressure (IOP) was similar between groups (14.31 ± 3.12 mmHg vs. 14.54 ± 2.36 mmHg, *p* = 0.898). CCT positively correlated with the length of hydroxychloroquine (HCQ) use (R: 0.357; *p* = 0.041), showing a trend toward an increase with prolonged usage, peaking approximately 100 months. Multivariate regression confirmed the association between SLE and higher CCT, potentially due to HCQ use.

**Discussion:**

We established an association between CCT and the presence of SLE, with SLE patients exhibiting significantly higher CCT values, potentially due to hydroxychloroquine use. These findings have important implications for IOP assessment, glaucoma risk evaluation, and refractive surgery planning in SLE patients and those undergoing treatment with HCQ. Further prospective studies are warranted to validate these observations and explore the underlying mechanisms.

## Introduction

Systemic lupus erythematosus (SLE) is a chronic autoimmune and multisystemic disease categorized as a “connective tissue disease.” In Europe, SLE is estimated to affect 39 out of every 100,000 individuals (1), with the highest prevalence observed among young women and individuals of Black, Asian, or Hispanic background (2). The European League Against Rheumatism (EULAR) and the American College of Rheumatology (ACR) have developed classification criteria for SLE, with a sensitivity of 96.1% and a specificity of 93.4% (3). However, not all patients meet these criteria, and many suffer from diagnostic delays and misdiagnosis.

SLE can potentially affect any tissue, organ, or system, with a preference for the musculoskeletal system, skin, and kidneys (4). Ocular involvement is also notable and can occur in up to 30% of patients (1, 5). These ocular manifestations, as noted by the British Isles Lupus Assessment Group (BILAG), are often excluded from the overall SLE activity assessment (1). Ocular abnormalities frequently correlate with systemic disease activity (5), yet they may go undetected because they can be asymptomatic or paucisymptomatic (1, 5), leading to treatment delay and a worse visual prognosis. This underscores the importance of ophthalmologic evaluation in both the diagnosis and monitoring of SLE.

The most common ocular manifestations in SLE are keratoconjunctivitis sicca due to secondary Sjögren’s syndrome (1, 6) and bilateral small vessel retinal vasculitis (1, 4, 6, 7). Other manifestations include recurrent corneal erosions, stromal corneal infiltration, peripheral ulcerative keratitis (PUK) (5), corneal edema (4), choroidal effusion, optic neuropathy (1), orbital or eyelid inflammation, and various retinal vascular abnormalities, such as vascular occlusions in relation to associated antiphospholipid syndrome (1).

Since the cornea is primarily composed of type I connective tissue (4, 6), it is particularly vulnerable to thinning and lysis phenomena in SLE due to inflammation caused by the deposits of immunocomplexes and autoantibodies (specifically anti-double-stranded DNA antibodies, anti-dsDNA, and anti-Smith antibodies, anti-Sm) in its basement membrane (4, 8). A limited number of studies have investigated the relationship between SLE and central corneal thickness (CCT), including those by Zang et al. (9), Çağlayan et al. (10), Yazici et al. (4), Mahendradas et al. (8), Eissa et al. (6), Kaya et al. (11), and Mahmoud et al. (5). However, these studies are significantly heterogeneous in their methodology, use of technology, inclusion criteria, and sample size, resulting in conflicting results. This results in uncertainty regarding CCT involvement in SLE, highlighting the need for further research.

In addition, medications commonly used to treat SLE, such as corticosteroids and hydroxychloroquine (HCQ), can have significant ocular side effects (1). The latest EULAR guidelines recommended HCQ for all SLE patients unless contraindication (12); consequently, most SLE patients are expected to be on prolonged HCQ therapy. HCQ can affect the cornea (13), leading to verticillate keratopathy (1) and alterations in endothelial cell density, and may result in CCT thickening (8, 10). It can also affect the ciliary body and retinal pigment epithelium (RPE), potentially resulting in maculopathy (1). Corticosteroids, meanwhile, increase the risk of both cataracts and glaucoma (1). Given these potential ocular complications, regular ophthalmologic monitoring is essential to mitigate the risk of vision impairment (1).

CCT measurement is essential for accurate intraocular pressure (IOP) assessment (14) and is a major source of error in applanation tonometry (15). A thicker cornea leads to overestimation of IOP; conversely, a thinner cornea results in underestimation (14). Accurate CCT evaluation is therefore crucial for diagnosing glaucoma, and also for diagnosing keratoconus and determining eligibility for corneal refractive surgery techniques such as Laser Assisted *In Situ* Keratomileusis (LASIK) or photorefractive keratectomy (PRK) (15–17).

Given the cornea’s susceptibility to thinning and lysis in SLE (4, 8) and the limited and inconsistent data regarding CCT in this population, further investigation is warranted. Based on the hypothesis that CCT is thinner in SLE patients compared to healthy individuals, this cross-sectional study aims to investigate the association between CCT and SLE by comparing mean CCT values between SLE patients and age- and sex-matched healthy controls using Zeiss HD Cirrus 5000’s optical coherence tomography (OCT).

## Materials and methods

This cross-sectional study was conducted at the Department of Ophthalmology, General University Hospital of Elda, in collaboration with the University of Miguel Hernández of Elche, Spain. The study protocol was previously published (18). Patient recruitment occurred between July 2020 and November 2024.

The study was conducted in compliance with all legal and ethical principles, including the Declaration of Helsinki (WMA, 2008) and the Good Clinical Practices of the European Union. Written informed consent was obtained from all participants. No patient identification data were included in the data collection form. Instead, a code was used and cross-referenced to identify data stored in a locked cabinet and only accessed by the principal investigator.

In accordance with current research legislation, the study protocol was approved by the Institutional Review Board (IRB) of the General University Hospital of Elda (PI2020/06).

Given the limited available literature, characterized by small sample sizes and conflicting findings, a pilot study including the first 20 patients (10 SLE patients and 10 healthy controls) was used to estimate the sample size. The mean CCT was 525 ± 25 μm in the SLE group and 510 ± 24 μm in the control group. Establishing a bilateral hypothesis with a type 1 error of 5% and a type 2 error of 20% (80% power), the resulting effect size was 0.61. This calculation indicated a minimum of 34 participants per group.

SLE patients were consecutively recruited from those referred by the Rheumatology Department to the Ophthalmology Department for HCQ macular toxicity screening. Medical records were reviewed to ensure the fulfillment of all inclusion and exclusion criteria. Control subjects were selected from those accompanying SLE patients to their appointments and individuals attending Ophthalmology consultations for occupational eye exams or minor ocular issues. Controls were recruited from the Health Department of Elda and matched by sex and age.

Patients were excluded in case of pregnancy or lactation, African descent, pre-existing systemic or ocular conditions potentially affecting corneal thickness (e.g., keratoconus, corneal edema, uveitis, diabetes mellitus, and chronic obstructive pulmonary disease), a history of glaucoma, severe astigmatism (> 3D), or severe myopia (≥ -6D or axial length > 26 mm). Those who regularly used contact lenses, had a history of ocular trauma or surgery, were using topical treatments other than artificial tears, had received topical or inhaled corticosteroid treatment in the past 3 months, or had received periorbital corticosteroids or systemic prednisone treatment at doses ≥7.5 mg/day in the past 6 months were also excluded.

Examinations were conducted between 9:00 AM and 2:00 PM to minimize diurnal variations in measurements. After obtaining written informed consent, a clinical interview and the following ophthalmologic tests were performed: refraction, visual acuity, slit-lamp examination of the anterior segment, fundus examination, pachymetry using Zeiss Cirrus HD-OCT 5000, Goldmann applanation tonometry (GAT), Schirmer 2 test, biometry (including keratometry), 24-2 visual field (VF), and OCT of the optic nerve with pupil dilation using tropicamide. Pachymetry was performed before the instillation of anesthetic and GAT to avoid tear film alterations or corneal deformations that could affect measurements. All ocular tests were performed in a single visit by a single observer, ensuring consistency between groups. Socio-demographic data, comorbidities, SLE manifestations, treatments, and laboratory results were also collected.

The Zeiss Cirrus HD-OCT 5000 was equipped with the HD Cornea lens. This attachment facilitates the generation of pachymetric maps divided into three concentric zones (0–2 mm, 2–5 mm, and 5–7 mm from the corneal center) by capturing 27,000 axial scans per second, with an axial resolution of 5 μm and a transverse resolution of 15 μm (19, 20). CCT measurements were obtained using the automated method (pachymetry map scan), based on 24 radial B-scans, each comprising 1,024 A-scans, covering a 9 mm diameter with a scan depth of 2 mm (20, 21). During the procedure, the patient’s gaze was fixed on the internal target, and the horizontal scan line was aligned with the corneal apex, ensuring proper positioning over the visible hyperreflective corneal reflex. Scans were repeated if the initial image was poorly centered or exhibited suboptimal corneal apex reflection. At least three corneal OCT scans were conducted per participant, and the image with the highest signal strength (≥60) and optimal quality was selected for analysis. For CCT measurements, the Cirrus HD-OCT software algorithm (version 10.0.0.14618) automatically identified the epithelium and endothelium boundaries, generating CCT map values for the three central corneal zones (19). The mean CCT value from the 0–2 mm central sector was used for analysis.

### Statistical analysis

Concordance analysis using the Kappa index demonstrated the perfect correlation between the right and the left eye (K = 1); therefore, one eye per participant was randomly selected for analysis.

Statistical analysis was performed using the Statistical Package for the Social Sciences (SPSS) version 26.0.0 developed by IBM Corp. (Armonk, NY) for Windows. The analysis was first conducted on the entire sample, followed by a subgroup analysis of SLE patients.

Quantitative data were expressed as mean ± standard deviation and categorical data as proportions. Normality was assessed using the Kolmogorov–Smirnov test. As the variables did not follow a normal distribution (*p* < 0.05), non-parametric tests were used. For group comparisons, quantitative variables were analyzed using the Mann–Whitney U-test, while categorical variables were assessed using the chi-square test for independent groups. McNemar’s test was used for dependent categorical variables, and Spearman’s Rho coefficient was used for correlation analysis. Loess regression was performed to visualize both linear and non-linear trends in the data. In addition, 95% confidence intervals (CI) were calculated for differences in proportions (categorical variable) and differences in means (quantitative variable) between the groups.

To minimize confounding bias and to adjust and evaluate potential interactions, multivariate linear regression was used to assess the relationship between CCT, SLE, and other factors. Both crude and adjusted coefficients were calculated for each variable. Statistical significance was defined as a *p*-value of <0.05. A stepwise variable selection process based on the Akaike information criterion (AIC) was performed for multivariate adjustment. Graphical analysis of goodness-of-fit was conducted, and no patterns suggesting heteroscedasticity, non-linearity, or non-normality were observed (Supplementary material 1).

## Results

The study involved 71 patients, aged 18 years or older, divided into two groups: 36 patients with SLE (Group 1) and 35 healthy controls (Group 2). Ten participants were male (14.1%), while 61 (85.9%) were female. The demographic and clinical characteristics of all participants are shown in Table 1 and details of the SLE group in Table 2.

**Table 1:** Socio-demographic and clinical characteristics of all study participants.

	Group 1	Group 2	*p*-value
Eyes No.	36	35	
Gender No. (%)			0.962
Female	31 (86.1%)	30 (85.7%)	
Male	5 (13.9%)	5 (14.3%)	
Age (years)			
Mean ± SD	45.8 ± 13.2	41.9 ± 12.7	0.059
Range	20–71	18–65	
RNFL No. (%)			0.666
Normal	33 (91.7%)	33 (94.3%)	
Abnormal	3 (8.3%)	2 (5.7%)	
VF No. (%)			0.321
Normal	35 (97.2%)	35 (100%)	
Glaucomatous	1 (2.8%)	0 (0%)	
Schirmer 2 No. (%)			0.361
Normal	30 (83.3%)	27 (77.1%)	
Abnormal	6 (16.7%)	8 (22.9%)	

Group 1: systemic lupus erythematosus group; Group 2: Control group. SD, standard deviation; RFNL, retinal nerve fiber layer; VF, visual field.

**Table 2:** Socio-demographic and clinical characteristics of the SLE group.

	Group 1 (SLE)
Raynaud (%)	
Yes	6 (16.7%)
No	30 (83.3%)
Ocular involvement (%)	
Yes	3 (8.3%)
No	33 (91.7%)
Musculoskeletal involvement (%)	
Yes	20 (55.6%)
No	16 (44.4%)
Mucocutaneous involvement (%)	
Yes	14 (38.9%)
No	22 (61.1%)
Renal involvement (%)	
Yes	3 (8.3%)
No	33 (91.7%)
Immunosuppressive treatment^a^ (%)	
Yes	3 (8.3%)
No	33 (91.7%)

a Patients under active treatment with methotrexate, mycophenolate, azathioprine, belimumab, leflunomide, or tacrolimus.

SLE, systemic lupus erythematosus.

The mean age in Group 1 was 45.8 ± 13.2 years, compared to 41.9 ± 12.7 years in Group 2, with no statistically significant differences between groups (*p* = 0.059). The mean duration of the SLE was 10.89 ± 8.9 years, and the mean time since the last SLE flare was 37.83 ± 36.4 months. All patients in Group 1 were treated with HCQ at a daily dosage not exceeding 6.5 mg/kg/24 h with a mean duration of HCQ usage of 81.79 ± 61.12 months. Three patients with SLE (8.3%) presented an altered retinal nerve fiber layer (RNFL), but only one patient (2.8%) demonstrated visual field defects and was diagnosed with glaucoma.

Table 3 summarizes the ocular tests for both study groups. The mean CCT in Group 1 was significantly thicker than in Group 2 (536.44 ± 39.91 μm vs. 517.57 ± 29.62 μm, *p* = 0.014), with a mean difference (MD) of 18.87 μm (95% CI: 2.58–36.45). No differences were observed in IOP between groups (14.31 ± 3.12 mmHg vs. 14.54 ± 2.36 mmHg, *p* = 0.898). A significant difference was found in the spherical equivalent (SE), with patients in Group 1 showing a more hyperopic refractive error than Group 2.

**Table 3:** Ocular measurements in all study participants.

	Group 1	Group 2	*p*-value
CCT (μm)			0.014
Mean ± SD	536.44 ± 39.91	517.57 ± 29.62	
Range	443–628	459–581	
IOP (mmHg)			0.898
Mean ± SD	14.31 ± 3.12	14.54 ± 2.36	
Range	6–20	10–21	
RNFL (μm)			0.637
Mean ± SD	92.89 ± 8.39	95.09 ± 9.86	
Range	66–108	74–116	
Sphere (D)			<0.001
Mean ± SD	0.74 ± 1.58	−1.06 ± 1.83	
Range	−2.5–4	−5.25–2.75	
Cylinder (D)			0.263
Mean ± SD	−0.76 ± 0.57	−0.67 ± 0.60	
Range	−2.75–0	−2.25–0	
SE (D)			<0.001
Mean ± SD	+0.47 ± 1.65	−1.31 ± 1.84	
Range	−2.75 – +3.75	−6.25–2.00	
Mean-K (D)			0.692
Mean ± SD	43.86 ± 1.19	43.92 ± 1.51	
Range	39.85–46.34	41.37–48.48	

CCT, central corneal thickness; SD, standard deviation; μm, microns; mmHg, millimeters of mercury; IOP, intraocular pressure; RFNL, retinal nerve fiber layer; SE, spherical equivalent; D, dioptric; Mean-K, mean keratometry.

**Figure 1:**
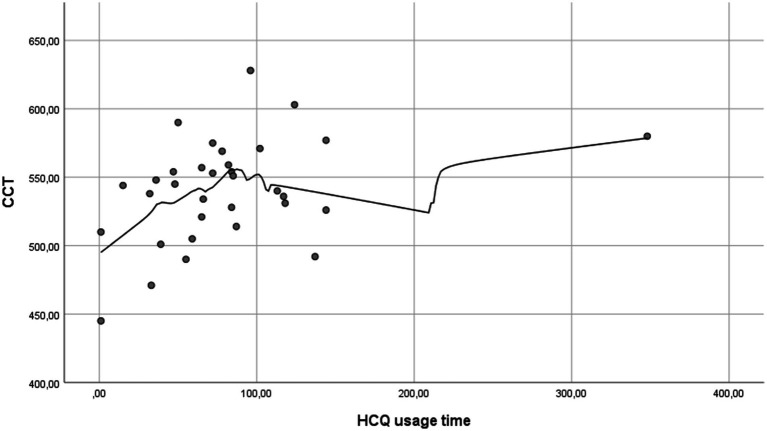
Simple scatter plot with Loess regression between CCT and HCQ usage time. CCT tends to increase with longer HCQ use, peaking approximately 100 months, after which it appears to stabilize (CCT, central corneal thickness; HCQ, hydroxychloroquine).

Table 4 presents the correlation analysis between CCT and several clinical variables. A moderate positive correlation was found between CCT and IOP (R: 0.307; *p* = 0.009) and between CCT and time of HCQ usage (R: 0.357; *p* = 0.041) (Figure 1). In addition, a moderate inverse correlation was detected between CCT and the presence of Raynaud’s phenomenon (R: −0.405; *p* = 0.014) and with previous mucocutaneous symptoms (R: −0.414; *p* = 0.012). No correlation was found between CCT and the duration of SLE (R: 0.102; *p* = 0.554) nor with the time since the last SLE flare (R: 0.199; *p* = 0.244).

**Table 4:** Correlation analysis of CCT and SLE characteristics.

		CCT
Duration of the SLE	R	0.102
	*p*-value	0.554
Time since the last SLE flare	R	0.199
	*p*-value	0.244
Time of HCQ usage	R	0.357
	*p*-value	0.041
Raynaud syndrome	R	−0.405
	*p*-value	0.014
Mucocutaneous involvement	R	−0.414
	*p*-value	0.012
IOP	R	0.307
	*p*-value	0.009

SLE, systemic lupus erythematosus; CCT, central corneal thickness; HCQ, hydroxychloroquine; IOP, intraocular pressure.

**Figure 2:**
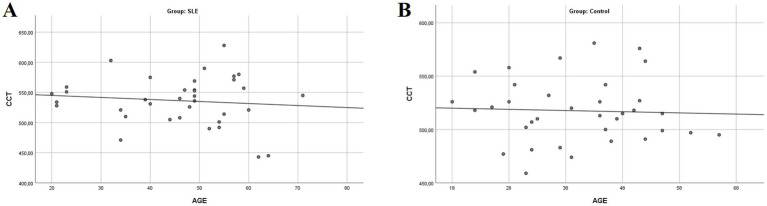
Simple scatter plot with linear regression between CCT and age in both groups. A trend of decreasing CCT with increasing age is observed in both groups, with a more pronounced decline in the SLE group (**A**: SLE group; **B**: control group; CCT, central corneal thickness).

Although there was no correlation between age and CCT (R: −0.011; *p* = 0.927), a trend toward decreasing CCT with increasing age was observed in both groups, with a more pronounced decline in the SLE group (Figure 2).

**Figure 3:**
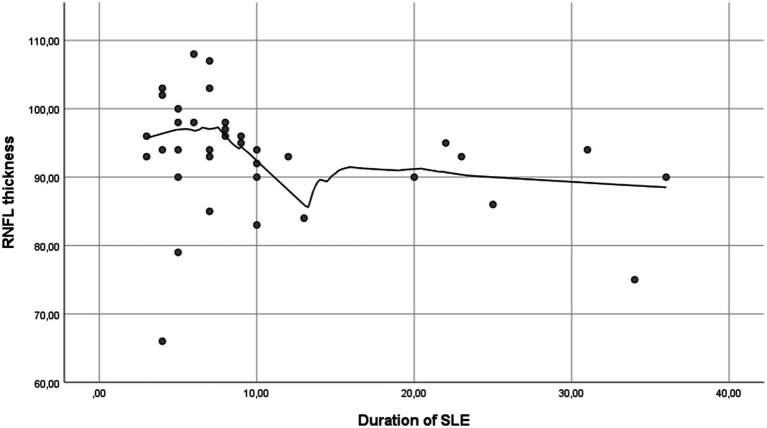
Simple scatter plot with Loess regression between RNFL thickness and duration of SLE. An inverse correlation between RNFL thickness and SLE duration is observed, indicating a trend of RNFL thinning over time (RNFL, retinal nerve fiber layer; SLE, systemic lupus erythematosus).

**Figure 4:**
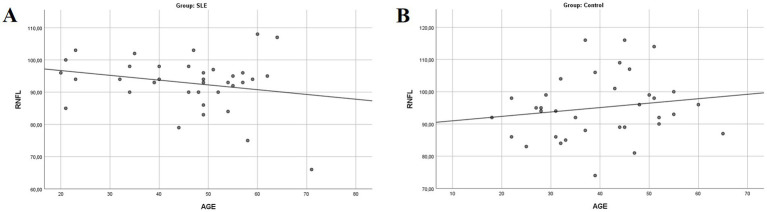
Simple scatter plot with linear regression between RNFL thickness and age (**A**: SLE group; **B**: control group; RNFL, retinal nerve fiber layer).

Furthermore, a moderate inverse correlation between RNFL thickness and the duration of SLE (R: −0.364; *p* = 0.029) was found (Figure 3), while no correlation was observed between RNFL thickness and age (R: −0.020; *p* = 0.868) (Figure 4). This finding suggests that SLE may contribute to the thinning of the RNFL, potentially indicating damage over time.

The multivariate linear regression analysis presented in Table 5 demonstrates an association between CCT and the presence of SLE in the unadjusted model (*p* = 0.027), which persisted after multivariate adjustment (*p* = 0.024), indicating that the effect of SLE on CCT was not influenced by other variables. Furthermore, the MD in CCT between the SLE and the healthy control groups increased to 19.61 μm after adjustment.

**Table 5:** Multivariate linear regression for CCT.

		Unadjusted			Adjusted Multivariate		
		Coefficient	Error	*p*-value	Coefficient	Error	*p*-value
Intercept		517.57	5.95	<0.001	1100.24	220.75	<0.001
SLE group	Yes	18.87	8.36	0.027	19.61	8.46	0.024
Sex	Woman				1.35	11.10	0.903
Age					−0.56	0.32	0.088
IOP					3.38	1.47	0.039
Sphere					NS		
Cylinder					NS		
Schirmer 2					NS		
RNFL					NS		

SLE, systemic lupus erythematosus; IOP, intraocular pressure; RFNL, retinal nerve fiber layer; NS, non-significant.

## Discussion

Ocular manifestations are present in up to 30% of patients with SLE (1, 5), with a special vulnerability of the cornea given its composition (4, 8). A limited number of studies have looked into the relationship between SLE and CCT (4–6, 8–11) reporting conflicting results.

Our study aimed to assess the relationship between CCT and SLE. This is of clinical relevance as changes in CCT can influence the diagnosis of several pathologies, as thinner CCT is an independent risk factor for glaucoma development (15). We identified an association between SLE and CCT, with SLE patients presenting a significantly thicker CCT compared to healthy controls (536.44 ± 39.91 μm vs. 517.57 ± 29.62 μm, *p* = 0.014). This association remained significant after adjustment (*p* = 0.024), with the multivariate linear regression revealing a mean difference (MD) increase in CCT from 18.87 to 19.61 μm.

Our findings align with the findings of Zhang et al. (9) and Çağlayan et al. (10). Zhang et al. did not directly measure CCT but rather corneal hysteresis (CH), inferring an association with CCT based on previous studies. They reported higher CH in SLE patients, which is positively correlated with CCT and suggests a thicker CCT in SLE patients (9). However, this contrasts with the findings of Gaspar et al. (16), who reported that the relationship between CCT and CH remains unclear and variable depending on whether the patients are glaucomatous or healthy (16).

Conversely, other studies such as Yazici et al. (4), Mahendradas et al. (8), Eissa et al. (6), Kaya et al. (11), and Mahmoud et al. (5) report a reduced CCT in SLE patients compared to controls (4–6, 8–11).

In our correlation analysis, we observed a moderate positive correlation between CCT and time of HCQ usage (R: 0.357; *p* = 0.041), with a trend of increased CCT with prolonged use, peaking approximately 100 months before stabilization. No correlation was detected between the duration of SLE nor the time since the last SLE flare (R: 0.102; *p* = 0.554 and R: 0.199; *p* = 0.244, respectively). These findings suggest that the observed increase in CCT with longer HCQ use is independent of the duration of SLE. Given that HCQ is commonly prescribed for SLE patients (3, 13, 22) and subject to ophthalmologic follow-up, several studies have investigated its long-term ocular effects. Çağlayan et al. found a significantly lower endothelial cell density (ECD) in HCQ-treated patients, with no differences in hexagonal cell percentage and no correlation between cumulative HCQ doses and ECD or CCT (10). These results are consistent with Oğurel et al., although only four patients with SLE were included in their study (23). Conversely, Vural et al. found no changes in ECD or CCT with HCQ usage but identified a negative correlation between the duration of HCQ use and corneal cell hexagonality (13). Consequently, the effects of HCQ on the cornea remain unclear, and further investigations are needed to elucidate the underlying mechanisms.

In addition, we found a moderate inverse correlation between the RNFL thickness and SLE duration (R: −0.364; *p* = 0.029), independent of age (R: −0.020; *p* = 0.868), suggesting that SLE may contribute to RNFL thinning, potentially increasing the risk of glaucoma. Furthermore, a moderate inverse correlation was identified between CCT and Raynaud’s phenomenon (R: −0.405; *p* = 0.014), which could indicate a heightened glaucoma risk in SLE patients with Raynaud’s, although no significant correlation was found between RNFL thickness and Raynaud’s phenomenon (R: 0.004; *p* = 0.983). The reported glaucoma prevalence in SLE patients in our cohort (2.8%) is similar to the published literature (3%) (24).

Glaucoma is the leading cause of irreversible blindness worldwide (25) and the second leading cause in Europe (26), with 111.8 million people expected to be affected by 2040 (25). Since glaucoma is typically asymptomatic until advanced stages, nearly 50% of cases go undiagnosed (26). While the etiology remains unclear, elevated IOP is the primary risk factor (16, 26), making accurate IOP and CCT evaluation crucial in clinical practice.

Precise and reproducible pachymetric measurements are essential, as a 40 μm decrease in CCT increases the relative risk of developing glaucoma by 1.71, and a 10% variation in CCT is associated with changes of 3–4 mmHg in IOP (15). Our study identified a moderate positive correlation between CCT and IOP, highlighting the potential for overestimated IOP values in SLE patients with increased CCT. This may contribute to diagnostic uncertainty when distinguishing true glaucomatous changes from those related to corneal thickening. Furthermore, the correlation between CCT and long-term HCQ therapy underscores HCQ’s impact on corneal properties, potentially contributing to CCT thickening and complicating IOP interpretation and glaucoma risk stratification.

SLE patients are also vulnerable to optic nerve damage. In our study, RNFL thinning was observed in three SLE patients, with one showing glaucomatous visual field defects leading to a glaucoma diagnosis. Notably, RNFL thinning was correlated with SLE disease duration, underscoring the importance of differentiating glaucomatous damage from optic nerve changes due to SLE progression.

Although ultrasound pachymetry (USP) is considered the gold standard for measuring CCT (19, 20), its use can be problematic. Accuracy requires the precise placement of the probe in the central cornea, with no movement or applied pressure. In addition, corneal hydration can influence measurements in USP (15). This leads to low reproducibility and potential inaccuracies (15, 19). The quantification of CCT through corneal OCT can offer advantages over USP, including faster measurement acquisition, a reduced need for patient cooperation, and eliminating the need for topical anesthesia, thus eliminating the risks of epithelial damage, corneal deformation, and potential ocular contamination (19). Corneal OCT has demonstrated a high reproducibility and is considered interchangeable with USP (19). Its high reproducibility, combined with its routine application in other ophthalmic assessments such as RNFL evaluation, makes OCT a practical and efficient tool for comprehensive eye care. Moreover, utilizing a single device for multiple assessments simplifies the diagnostic process and aligns with the increasing demand for efficient, less invasive, patient-centered care.

Our research aims to improve methodological rigor compared to previous studies by determining representative sample size and exclusively enrolling SLE patients to enhance participant homogeneity. Several prior studies exhibited substantial variability in sample sizes and provided no *a priori* sample size calculation. For instance, the studies by Mahendradas et al. and Oğurel et al. included small samples of seven and four SLE patients, respectively (8, 23), limiting their analyses to descriptive statistics, similar to Mahendradas et al. (8), or necessitating the inclusion of both eyes in the analysis, as done by Mahmoud et al. (5). In addition, some studies such as those by Mahendradas et al. (8) included patients with both active and inactive SLE. In contrast, others, such as Yazici et al., Zhang et al., and Mahmoud et al., did not specify the SLE activity status (4, 5, 9). In contrast, Eissa et al. focused exclusively on active SLE patients (6), whereas the studies by Kaya et al. and Çağlayan et al. examined only patients with inactive SLE (10, 11).

Moreover, we ensure the correct diagnosis of SLE because the patients were diagnosed and referred by a rheumatologist, which guarantees its accuracy and reliability. In addition, a concordance analysis was performed using the kappa index to assess the consistency of measurements in both eyes, resulting in a perfect correlation (K = 1) between both eyes. Due to this, only one eye per patient was included; the eye to be included was randomly selected, contrary to previous investigations, where the choice of the eye analyzed was chosen by convention without exploring whether there were differences between both eyes. Furthermore, to minimize potential confusion bias and possible interactions, a multivariate analysis was performed. To minimize measurement bias, primary outcome data were collected through clinical interviews and tests using calibrated and validated devices. The main study variable depended on an objective test, and all participants, regardless of their assigned group, were evaluated in the same manner by the same investigator, ensuring no differences in assessments between groups.

However, our study presents some potential limitations. First, the initial uncertainty about the appropriate sample size required a pilot study, and the consecutive sampling method may limit the representativeness and generalizability of the conclusion. Second, the cross-sectional study design lacks a temporal sequence, thereby demonstrating association but not causality. Another consideration is that Zeiss Cirrus HD-OCT 5000 measures CCT from the tear film to Descemet’s membrane (19), potentially reducing accuracy in patients with ocular surface disorders, such as low tear production or a diminished tear meniscus. To mitigate this, the Schirmer 2 test identified at-risk patients, and preservative-free artificial tears were applied to all participants before pachymetry. Nevertheless, the Cirrus HD-OCT review software automatically identified and measured corneal epithelial thickness as the distance between the midpoint of the first hyperreflective line, corresponding to the tear film, and the second hyperreflective line, representing the anterior surface of Bowman’s membrane. This methodology minimizes the inclusion of the tear film in the CET measurement, ensuring more precise results (20). Finally, given the absence of an initial CCT evaluation, the temporal stability of CCT once the SLE has been diagnosed is unknown, and the unexpected results contradicting our hypothesis regarding CCT decrease in SLE may be attributed to HCQ usage and its corneal deposition. To address these issues, a prospective study will be conducted to reassess CCT in SLE patients 5 years after baseline CCT examination.

It is also important to acknowledge a potential selection bias among the healthy group, as participants are selected based on the absence of an established SLE diagnosis, without additional confirmation through complementary tests. However, according to the Spanish Society of Rheumatology’s EPISER study, which included 4,900 participants to determine the national prevalence of different rheumatic diseases in Spain, 12 cases of SLE were identified through a telephone interview screening. Out of these, 11 cases had an established diagnosis before the screening, with only one patient being diagnosed *de novo* (27). Given the estimated low prevalence of SLE, the likelihood of misclassifying patients without self-reported or documented SLE in their medical history is low.

Understanding the association between CCT and SLE could enhance ophthalmologic care and improve the quality of life for patients. These findings underscore the importance of incorporating CCT evaluation into the routine ophthalmologic assessment of SLE patients, particularly in the context of glaucoma screening and systemic disease monitoring. Collaboration between ophthalmologists, rheumatologists, and researchers is crucial for exploring underlying mechanisms, such as inflammation, immune response, and the role of HCQ. This multidisciplinary approach could shed light on the behavior of the cornea in the context of SLE. However, further research is needed to fully elucidate the underlying mechanisms and their clinical implications.

## Conclusion

In conclusion, we established a significant association between CCT and the presence of SLE, with SLE patients showing significantly higher mean CCT values compared to controls. This increase may be attributed to long-term HCQ use, as CCT shows a rising trend with prolonged HCQ usage, peaking approximately 100 months before stabilization. These findings have important clinical implications for IOP assessment, glaucoma risk evaluation, and refractive surgery planning in SLE patients and those on HCQ therapy. Further prospective studies are warranted to validate these observations and explore the underlying mechanisms.

## Data Availability

The raw data supporting the conclusions of this article will be made available by the authors without undue reservation.
