# Supplementary material for: Analysis of central corneal thickness in systemic lupus erythematosus
Source: Front Med (Lausanne). 2025 Feb 28;12:1545415. doi: 10.3389/fmed.2025.1545415 (PMC11907372; doi:10.3389/fmed.2025.1545415)

**SUPPLEMENTARY MATERIAL 1:** Graphical analysis to detect lack of normality, heteroscedasticity, or lack of linearity.


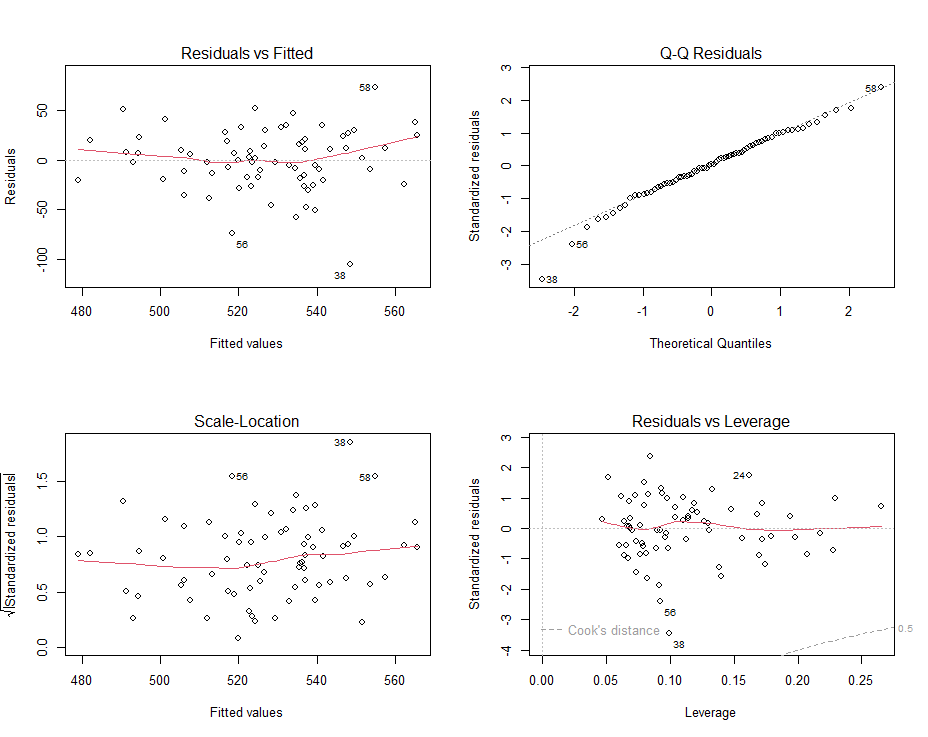

Supplement: Supplementary file 1 [file Table_1.DOCX]
